# Dislocation-mediated relaxation in nanograined columnar palladium films revealed by on-chip time-resolved HRTEM testing

**DOI:** 10.1038/ncomms6922

**Published:** 2015-01-05

**Authors:** M. -S. Colla, B. Amin-Ahmadi, H. Idrissi, L. Malet, S. Godet, J. -P. Raskin, D. Schryvers, T. Pardoen

**Affiliations:** 1 Institute of Mechanics, Materials and Civil Engineering, Université catholique de Louvain, Place Sainte Barbe 2, B-1348 Louvain-la-Neuve, Belgium; 2 Department of Physics, Electron Microscopy for Materials Science (EMAT), University of Antwerp, Groenenborgerlaan 171, B-2020 Antwerp, Belgium; 3 4MAT: Materials Engineering, Characterization, Synthesis and Recycling, Université Libre de Bruxelles, 50 Avenue FD Roosevelt CP194/03, 1050 Brussels, Belgium; 4 Information and Communications Technologies, Electronics and Applied Mathematics (ICTEAM), Université catholique de Louvain, Place du Levant 3, B-1348 Louvain-la-Neuve, Belgium

## Abstract

The high-rate sensitivity of nanostructured metallic materials demonstrated in the recent literature is related to the predominance of thermally activated deformation mechanisms favoured by a large density of internal interfaces. Here we report time-resolved high-resolution electron transmission microscopy creep tests on thin nanograined films using on-chip nanomechanical testing. Tests are performed on palladium, which exhibited unexpectedly large creep rates at room temperature. Despite the small 30-nm grain size, relaxation is found to be mediated by dislocation mechanisms. The dislocations interact with the growth nanotwins present in the grains, leading to a loss of coherency of twin boundaries. The density of stored dislocations first increases with applied deformation, and then decreases with time to drive additional deformation while no grain boundary mechanism is observed. This fast relaxation constitutes a key issue in the development of various micro- and nanotechnologies such as palladium membranes for hydrogen applications.

## Abstract (web summary)

Nanostructured metallic materials involve a high rate sensitivity usually resulting from grain boundary related mechanisms. Here, the authors report mechanical tests on freestanding Pd thin films and show that creep is associated with dislocations rather than grain boundaries.

## Graphical abstract

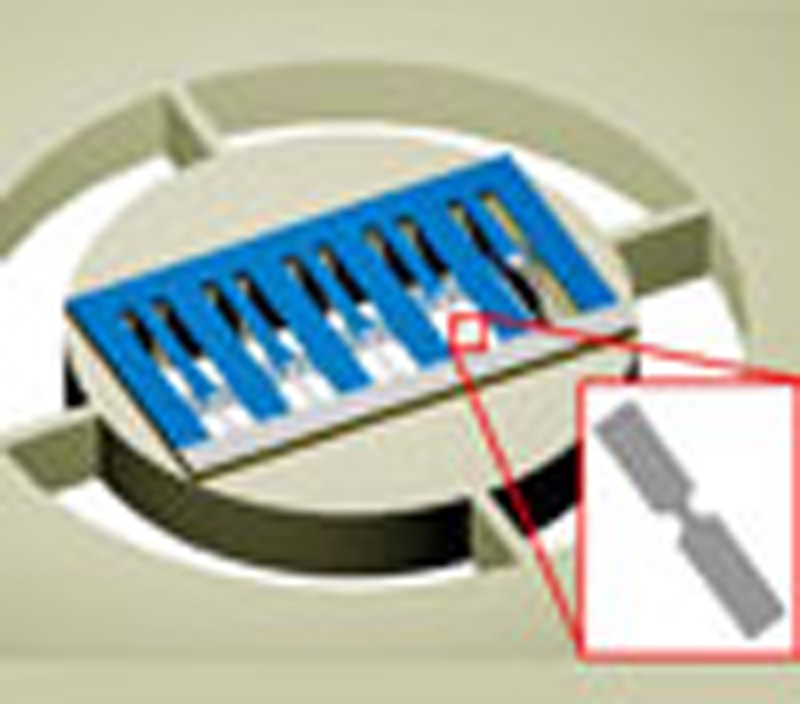

Nanocrystalline metals present excellent mechanical performance in terms of strength and fatigue resistance but often at the expense of ductility^1^. Furthermore, nanocrystalline systems show moderate to high rate sensitivity at room temperature^2^, which might help restoring the ductility^3^ but can lead to detrimental creep/relaxation effects in applications. The high-rate sensitivity results from a variety of thermally activated mechanisms involving grain boundary (GB) sliding, diffusion, grain growth and dislocation nucleation^1^,^4^,^5^,^6^. Some of these mechanisms are well understood; however, the interactions among them in terms of possible cooperation or competition constitute a matter of open research. Thin metallic films constitute ideal candidates for looking at the mechanics of columnar-grained and -textured nanocrystalline systems as they can be easily produced with nanograined structures frequently involving a single grain along the growth direction and sharp crystallographic textures^7^. The small thickness allows for direct *in situ* testing by transmission electron microscopy (TEM) to characterize deformation mechanisms^8^,^9^. In addition to fundamental interest, thin nanocrystalline metallic films are essential constituents of microelectronics, microelectromechanical systems (MEMS), functional coatings or membrane applications.

Different methods have been used to characterize the rate-sensitive creep or relaxation behaviour at the nanoscale, involving nanoindentation, bending and direct tensile testing^4^,^10^,^11^. The main shortcomings involve the difficulty to impose very small strain rates typical of real applications and to perform *in situ* relaxation tests in order to characterize the deformation mechanisms. In addition, the sensitivity to drift is increased at nanoscale, which prevents imposing a constant load during long periods of time. A technique consisting of the measurement of the stress evolution during thermal cycling and allowing *in situ* observation has been proposed but the interpretation and high-resolution observations are complicated^12^.

Here palladium (Pd) films are studied as a reference system to investigate hardening and relaxation in nanocrystalline materials with growth nanotwins, as well as for its importance in hydrogen technologies such as in sensors or membranes. We report time-resolved high-resolution TEM (HRTEM) creep/relaxation experiments using an on-chip testing method. Note that, as images cannot be continuously recorded and as the TEM equipment cannot be monopolized for several days in a row, the specimens had to be stored outside the microscope during the waiting times between two series of observations. The study is thus not strictly speaking *in situ*, but could be fully *in situ* if relaxation was faster. The films involve ~30-nm grain size and initially contain perfectly coherent twin boundaries (TBs). Once deformed, the TBs lose coherency because of intense dislocation activity. During relaxation, dislocations keep accumulating at TB, while the total dislocation density decreases. The activation volume of the Pd films extracted from the stress–strain evolution is ~20 *b*^3^ and increases during relaxation as a result of the decrease in dislocation density. In the following, we demonstrate that dislocation-mediated mechanisms dictate the mechanical response of Pd films during both deformation and relaxation, despite the nanosized grain structure.

## Results

### Mechanical characterization by on-chip testing

**Figure 1:**
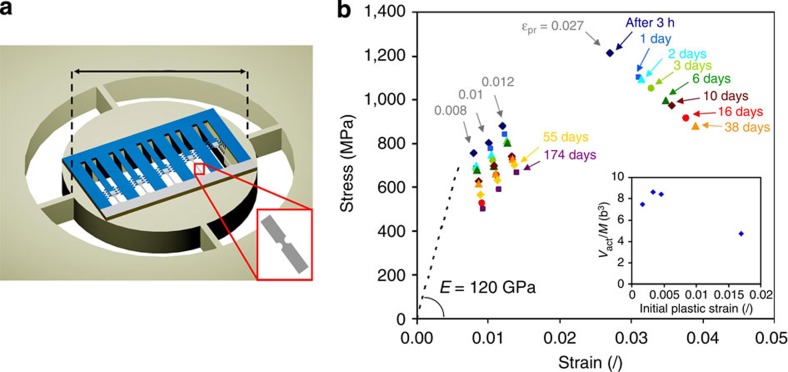
On-chip testing method for mechanical characterization. (**a**) Schematic view of the structures allowing time-resolved TEM observation with 3-mm scale bar. Notches have been milled at every two beams using FIB (see Methods for more details). (**b**) Stress–strain evolution of different Pd beams under uniaxial tension. The Young’s modulus has been determined by nanoindentation as equal to 120 GPa. The initial strain level *ε*_pr_ before relaxation is indicated in the figure. The inset presents the mean activation volume evolution as a function of the initial strain *ε*_pr_ present in the different beams before relaxation. The activation volume *V* decreases when the initial strain level increases.

An on-chip testing method has been used to determine the mechanical response of 90-nm-thick Pd film under uniaxial tension. The design of the testing method allows time-resolved TEM observation of the films under deformation and relaxation (see Fig. 1a and Methods). The mechanical analysis considers straight beams without notches only. The stress and strain evolution during relaxation is shown in Fig. 1b. The fracture strain is ~3% and the yield stress ~500±60 MPa close to the internal stress measured after deposition. The reproducibility of the measurements has been verified by testing different series of tensile stages from the same batch (Supplementary Fig. 1).

### As-deposited microstructure of the Pd films

**Figure 2:**
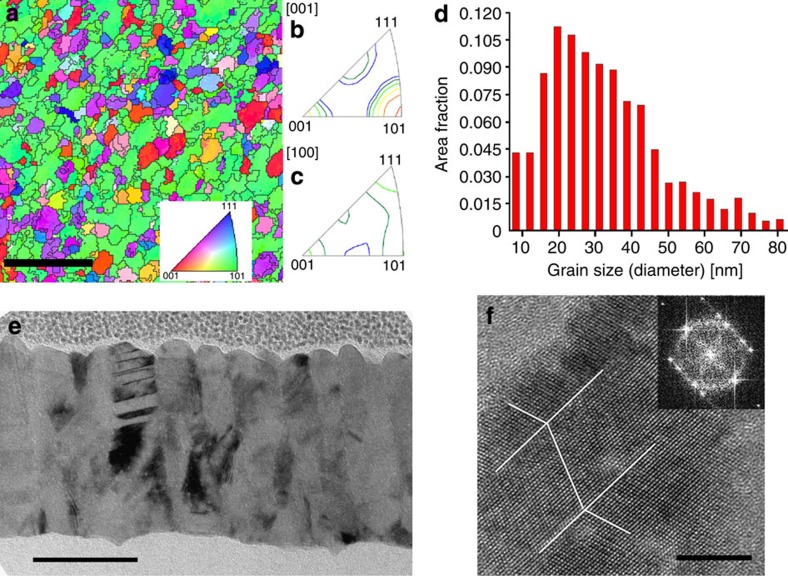
Microstructure characterization of the as-deposited Pd film. (**a**) ACOM-TEM orientation mapping of a plan-view FIB sample with 200-nm scale bar. The film has been thinned to obtain one grain through the thickness. (**b**) Inverse pole figure showing the {110} preferential orientation of the grains in the direction perpendicular to the film. (**c**) Inverse pole figure showing the random orientation of the grains in the direction parallel to the film, that is, in the tensile direction. (**d**) Grain size distribution of the as-deposited Pd film obtained from the ACOM-TEM map presented in **a**. The grain size distribution has been determined using a grain tolerance angle of 10°. (**e**) Bright field micrograph obtained on cross-sectional FIB sample with two or three grains over the thickness with 50-nm scale bar. (**f**) HRTEM image showing a Σ3 60° {111} growth nanotwin with perfectly coherent TBs with 5-nm scale bar.

The microstructure and texture of the as-deposited Pd films evaporated at 1 Å/s were characterized using both cross-sectional and plan-view foils prepared by focused ion beam (FIB; see Fig. 2 and Methods). The structure is columnar with 2 or 3 grains over the thickness and an in-plane grain diameter of ~30 nm. The dislocation density in the as-deposited film measured by HRTEM is equal to 4±0.7 × 10^16^ m^−2^ as an average from four grains (for details about the HRTEM and the counting of dislocations see the Methods part, Supplementary Note 1 and Supplementary Fig. 2). The microstructure involves Σ3 60° {111} coherent growth nanotwins in ~25% of the grains^13^,^14^. A fibre texture is observed with the [110] direction oriented perpendicular to the film and no in-plane preferential orientation.

### Dislocation density evolution

**Figure 3:**
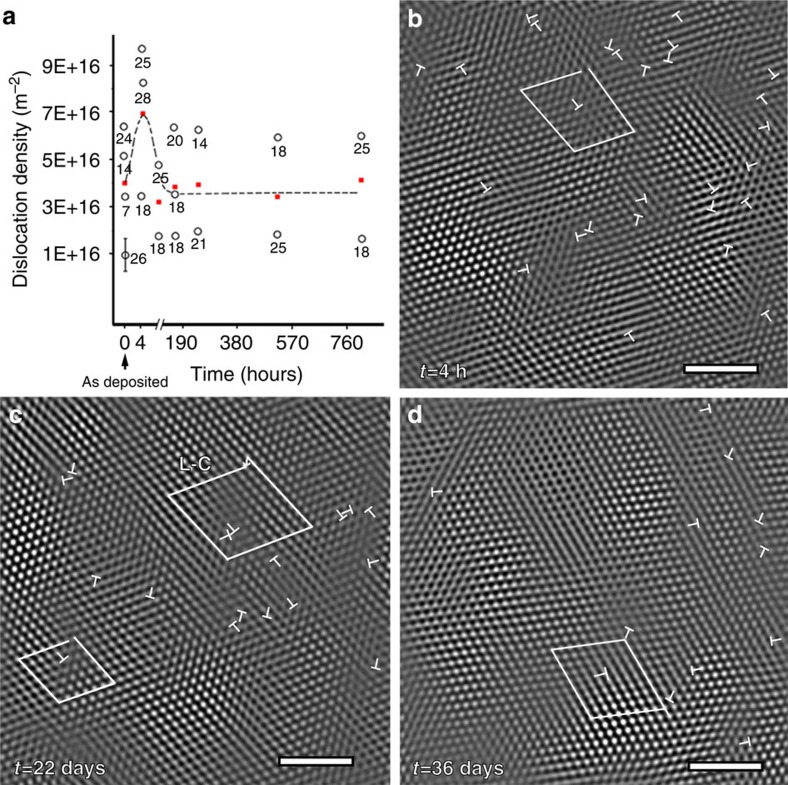
Dislocation density evolution upon relaxation. (**a**) Average dislocation density (red solid squares) evolution of the Pd film versus time (open circles are measurements of individual grains with listed numbers indicating the corresponding grain size (nm)). The average dislocation density in as-deposited Pd films is 4±0.7 × 10^16^ m^−2^. On the images, the time after the release is given in hours. Dislocation density first increases when deformation is applied after release and then decreases upon relaxation. (**b**–**d**) Filtered HRTEM images showing the continuous change of the dislocation positions with time. Note the formation and destruction of Lomer–Cottrell dislocations indicated by L-C in **c**. The perfect dislocations are indicated by ‘T’ symbols. The scale bar is 2 nm.

**Figure 4:**
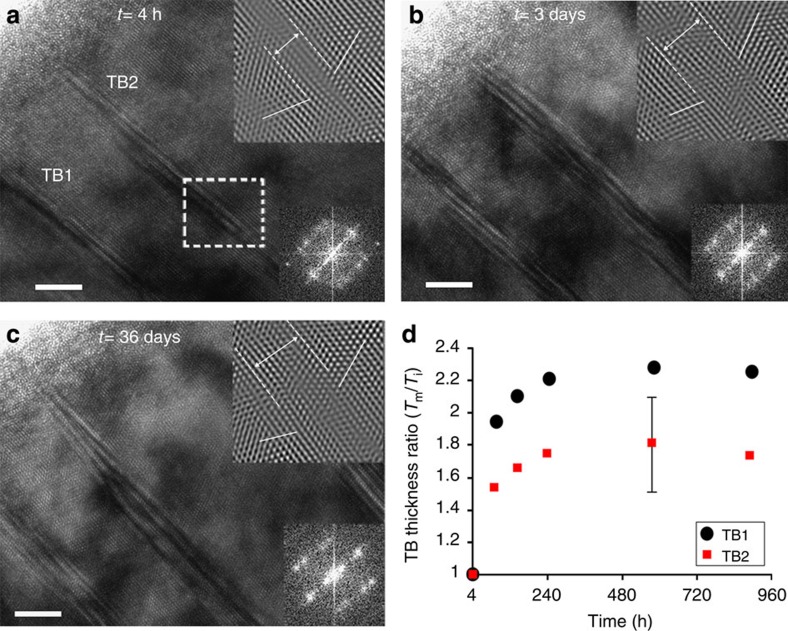
TB thickness evolution upon relaxation. (**a**–**c**) HRTEM images showing Σ3 {111} TBs at *t*=4 h, 3 and 36 days after release. Note the increase in the TB thickness from **a**–**b** in the filtered images at the upper right insets of the micrographs, with 5-nm scale bar. No significant change of the TB thickness from **b**–**c** is observed. (**d**) Evolution of the TB thickness ratio (*T* measured/*T* initial) of the TB1 and TB2 in **a**. A plateau is reached after 10 days.

The first TEM observation of deformed specimens has been performed 4 h after release. These samples are observed under plan-view conditions with the Pd beam still being intact (except for the FIB-induced notches) and with the substrate and sacrificial layer being removed to allow electron transparency. This implies that no images of the pristine, that is, the unreleased sample, are available for the data shown in Figs 3 and 4. The mean dislocation density measured as an average from different grains deformed by ~2% increases up to 7±0.7 × 10^16^ m^−2^ (Fig. 3a), which is almost twice the initial density found in the as-deposited film. The scatter in dislocation density is caused by the local nature, that is, individual nanocrystalline grains, of the HRTEM measurements. This dislocation density is more than one order of magnitude larger than the dislocation density usually measured in nanocrystalline materials^15^,^16^,^17^. It is commonly claimed that nanograin sizes prevent dislocation storage. Recent investigations also found significant dislocation accumulation in nanocrystalline Ni^15^,^17^ and Pt^16^. The presence of pre-existing dislocations and twins in the as-deposited films presumably favours additional storage by providing pinning sites (see further). Only perfect dislocations were observed with no stacking faults or deformation twins during deformation, in agreement with the high stacking fault energy of Pd (*γ*_sf_≈180 mJ m^−^^2^) and confirming previous *ex situ* TEM characterizations performed on the same Pd films^18^. Furthermore, twinning/detwinning due to twin boundary migration in grains containing pre-existing growth twins has not been observed in the present work. In Pd, the grain size under which partial dislocation emission is favoured over full dislocation emission is ~13 nm (ref. 5).

During relaxation, the dislocation density decreases by a factor ~2 to reach a steady-state level (Fig. 3a). In Figure 3b–d, the inverse fast Fourier transform (IFFT) images extracted from HRTEM images by selecting all three first order **g**-vectors 
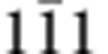
, 
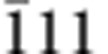
 and 002 illustrate the dislocation activity during relaxation at exactly the same location over time. Note that the slight rotation of the HRTEM images in Fig. 3b–d is because of the global rotation of the sample during the successive insertion/removal of the TEM holder in the microscope over the different days of measurement. The positions of perfect dislocations lying in two different {111} slip planes are continuously varying (see *T* symbols added for all observed dislocations in Fig. 3b–d). Interestingly, Lomer–Cottrell dislocations have been detected. This kind of sessile dislocation is very effective to induce hardening and is typical of face-centred cubic metals with medium to high stacking fault energy^15^, such as Pd. More surprisingly, the Lomer–Cottrell locks disappear with time (see Fig. 3b–d, Supplementary Note 2 and Supplementary Fig. 3). Recently, a similar formation and breaking of Lomer–Cottrell junctions has been observed in Pt films with 10-nm grain size^16^. Numerical simulations predict that a stress of ~750 MPa is needed to destroy the junction in Pd^19^, which falls in the range attained in this study. Hence, despite the 30-nm grain size, dislocation-based mechanisms dictate the mechanical response of the Pd films during both the fast predeformation and the slow relaxation. Note that the local time-resolved evolution of dislocation density measured in the region shown in Fig. 3b is in agreement with the results of Fig. 3a coming from an average over multiple grains (see Supplementary Fig. 4).

### TB morphology evolution

TBs constitute strong barriers to dislocation motion. Initially, in the as-deposited film, most of the Σ3 60° {111} TBs are perfectly coherent, see Fig. 2f. However, the coherency is lost during deformation due to the interaction of lattice dislocations with the TBs^13^,^18^. The loss of coherency of these TBs keeps increasing upon relaxation as indicated by the progressive increase in the TB1 and TB2 thickness (Fig. 4). This is due to the formation of residual interfacial defects at the TBs such as Frank sessile dislocations^13^,^18^ and the accumulation of lattice dislocations^20^. Further, HRTEM observations have confirmed the behaviour shown in Fig. 4d, see Supplementary Note 3 and Supplementary Fig. 5. Note that the evolution of ‘TB thickness’ should not be confused with a possible evolution of the ‘twin thickness’, which can occur by TB migration induced by the nucleation and glide of twinning dislocations. The latter, however, was not observed in the present work.

The term ‘TBs thickness’ is defined here as the distance separating the two last nondistorted twinning planes delimiting matrix and twin. The positions of these planes are indicated by dashed white lines in the upper right insets of Fig. 4a–c, from which the inclined {111} planes indicated by white solid lines in the same insets start to deviate from the initial position because of the accumulation of dislocations at the TBs (see Supplementary Note 3 and Supplementary Fig. 6). Because the thickness may change along individual TBs, the measurements reported in Fig. 4d were always performed in the same region indicated by dashed lines in Fig. 4a and shown in the upper right insets of Fig. 4a–c. In order to ensure HRTEM observation of TBs in as identical local orientation conditions as possible, the intensity symmetry of the FFT patterns was accurately compared (see the lower right insets of Fig. 4a–c). The presence of twins clearly participates to the increased dislocation storage capacity of nanotwinned metals^21^, as evidenced by the progressive loss of coherency of TBs. This study thus shows, as an original side result, that Σ3 {111} coherent TBs can be used as markers of the level of plastic deformation developed in individual nanograins during creep.

### Evolution of grain size distribution

Another set of test structures was used for ACOM-TEM measurements to provide statistical data on the grain size distribution variation upon relaxation (Supplementary Fig. 7). The ASTAR software was used for the data acquisition. No evolution of grain size is observed, confirming the absence of GB migration. No GB sliding has been observed by conventional TEM analysis. Hence, there are strong lines of evidence that no GB-type mechanisms contribute to relaxation in the present <110> textured columnar grained specimens. Obviously, this specific columnar microstructure may affect the importance of GB processes and further experimental and modelling studies are required to better understand the impact of these morphological features on the competition between the different deformation mechanisms.

### Strain-rate-sensitivity analysis

The strain-rate sensitivity has been evaluated from the stress versus strain evolution following the approach proposed in ref. 22 in order to provide more quantitative insight into the relaxation mechanisms related to the TEM observations. The strain-rate-sensitivity exponent *m* is defined as 
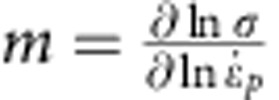
 and the activation volume *V* is defined as 
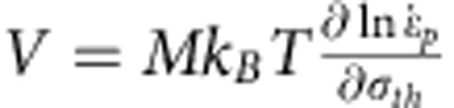
, where *σ*_th_ is the thermally activated component of the stress, 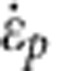
the plastic strain rate, *M* the Taylor factor, *k*_B_ the Boltzmann constant and *T* the temperature. The activation volume is a signature of the dominating thermally activated deformation mechanism. The activation should, in principle, be evaluated under conditions of frozen microstructure, using strain-rate jump tests^23^. Here only an apparent activation volume can be extracted because of the slow nature of the tests. The test gives access to the total resistance, only which combines the thermal component and a long-range contribution. Details of the procedure used to determine the true activation volume based on the apparent value are given in the Supplementary Note 4. Although only semiquantitative, the analysis is sufficient for our purpose as the conclusions remain the same if the correction on the data changes by a factor of two or more. The values reported hereafter correspond to the true activation volume after correction.

The inset of Fig. 1b shows the variation of *V*/*M* as a function of the plastic prestrain. This value is an average over the time of relaxation, see the Methods part. If *M* is taken equal to √3 as proposed for face-centred cubic nanocrystalline materials^5^, *V* is between 17 and 29 *b*^3^. These values agree with the values reported in literature for nanotwinned Cu^10^ and nanocrystalline Ni^4^. These values are larger than what is expected for diffusion (~1 *b*^3^) or dislocation nucleation (a few *b*^3^)-dominated deformation mechanisms. The activation volume decreases with increasing plastic prestrain. This agrees with the idea that *V* depends on dislocation density *ρ*. Taking *V* as proportional to the spacing between two pinning points, *V* should be inversely proportional to √*ρ* (see Supplementary Note 5), in agreement with ref. 12, if there are, on average, more than a few dislocations in each grain, which has been confirmed with TEM. In the absence of dislocation, *V* remains constant for a given grain size. For the dislocation density of 7±0.7 × 10^16^ m^−2^ attained after 2% predeformation, the corresponding *V*~20 *b*^3^ matches with the experimental values. Using a more sophisticated derivation procedure where *V* is not taken *a priori* as constant, see Methods, *V* tends to increase by a factor 3–10 over 38 days of relaxation. This increase can thus be associated to a decrease in *ρ*, again in accordance with the experimental observations, see Fig. 3a. The increase in the activation volume during relaxation can be quantitatively related to the decrease in the dislocation density with time using simple arguments developed in Supplementary Note 6. Hence, the time to restore dislocations pinned by other dislocations and junctions constitutes the most probable mechanisms dominating the relaxation behaviour, in agreement with the TEM observations. Note that additional effects probably contribute to the evolution of *V* such as the loss of coherency of the TBs that can change the time of interaction of the dislocations meeting such TBs.

## Discussion

The on-chip testing technique developed in this work in combination with time-resolved observation of the relaxation mechanisms yields data difficult to obtain using classical methods during long periods of time. The technique is compatible with fully *in situ* analysis in case of faster relaxation rates or if an external perturbation is added to slightly modify the load equilibrium condition such as with a small temperature change. The observation that dislocations can dominate both the hardening and relaxation behaviours in the present <110> textured columnar nanocrystalline materials even at 30-nm grain size opens perspectives to control the mechanical properties of nanostructured materials. For instance, GB-mediated mechanisms that often lead to a high-rate sensitivity of nanocrystalline materials can be avoided or, at least, mitigated, by the presence of a <110> textured columnar microstructure. Still, the relaxation mechanisms observed in this study can be detrimental to the mechanical stability in applications involving Pd-based nanobeams or membranes.

## Methods

### On-chip nanomechanical testing of Pd films

This part is inspired by the supporting information of ref. 13. The on-chip nanomechanical testing method used in this work to measure the tensile response of thin Pd films relies on microfabrication techniques as developed for microelectronic devices and MEMS. The concept of the method^22^,^24^ is to use the internal stress (or equivalently the elastic mismatch strain) present in a long beam, ‘the actuator’ (here 30-nm-thick Si_3_N_4_), to deform another material attached to it, ‘the specimen’ (here 90-nm-thick, ~2-μm-wide Pd ribbons; Supplementary Fig. 8). The actuator material is selected in order to involve large internal stress building up during deposition. The actuator and the specimen are patterned by successive photolithographies, and lie on a SiO_2_ sacrificial layer, deposited on Si substrate. The Si wafer is selectively etched from the backside to enable direct in-plane TEM observation (Fig. 1a). The SiO_2_ sacrificial layer is finally etched leading to the actuator contraction and deformation of the Pd beams under uniaxial tension (Supplementary Fig. 8). The stress *σ* and the mechanical strain *ε*^mech^ in the deformed Pd specimens are provided by the measurement of the specimen elongation and from knowing the magnitude of the mismatch strain of both the actuator 
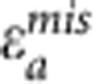
 and test sample *ε*^mis^.

The mechanical strain in the test specimen and actuator is given by the difference between the total and mismatch strain, that is


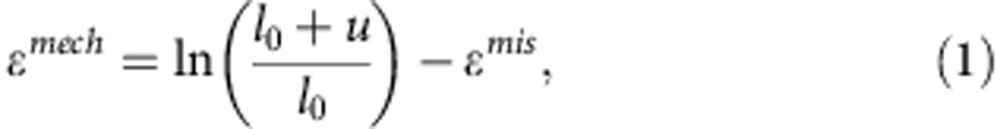



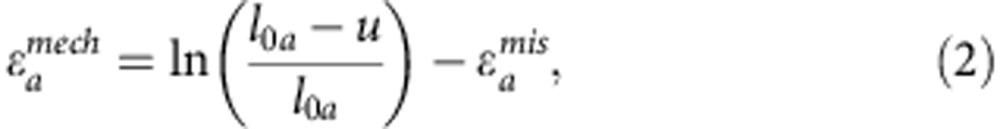


respectively, where *l*_0_ is the initial length of the specimen, *l*_0a_ is the initial length of the actuator beam and *u* is the displacement of the structure. Assuming the actuator material remains elastic and knowing the Young’s modulus, the stress is given by


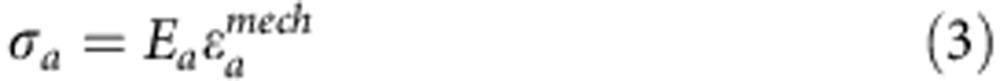


where *E*_a_ is the Young’s modulus of the actuator material. The actuator is made here of Si_3_N_4_, which is a brittle linear elastic material. Finally, the stress in the specimen can be calculated using


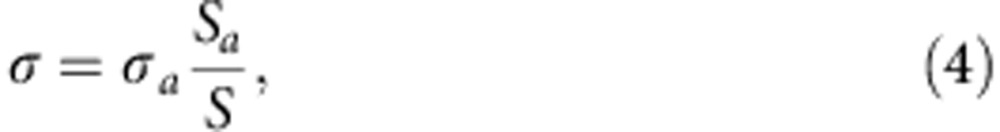


where *S*_a_ and *S* are the actuator and test specimen cross-section areas, respectively.

A complete stress–strain curve is generated by varying the actuator versus specimen length ratio (see Supplementary Fig. 9a). The design and error analysis have been detailed in ref. 24. The most important sources of error on the stress extraction come from the errors on the measurement of *u* and 
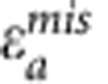
. The uncertainty on the displacement measurement is considered to be better than 50 nm when using high-magnification field emission gun scanning electron microscope (FEG SEM) micrographs (Supplementary Fig. 9b and c). The current precision estimation of the mismatch strain is about 5% using dedicated test structures near the set of tensile structures, such as rotating sensors^25^,^26^, free actuators and clamped–clamped beams^27^. This leads to an error on the stress of ~10%. The value of the mismatch strain is also cross-checked based on Stoney-type measurement of the internal stress combined with the knowledge of the Young’s modulus obtained by nanoindentation^27^. The value of 
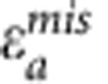
 is typically ~0.003. Regarding the measurement of the strain, only the error on *u* is critical. Considering the dimensions of the test structures, it results in less than 5% relative error on the strain.

The main advantage of the method is the capacity to very easily produce a high number of data, the simplicity and the capacity to generate well-controlled stress state. By extracting a stress–strain curve from different specimens, the method takes into account material scatter. The main disadvantage is that the loading rate is uncontrolled and that the specimen is always analysed within the relaxed configuration.

### Fabrication process

A double-side-polished Si wafer with a thickness of 180±20 μm was selected. First, a 1-μm-thick plasma-enhanced chemical vapour deposition SiO_2_ sacrificial layer was deposited on the front side of the wafer. This layer was densified during 20 min at 800 °C to provide optimum control of the etching rate (that is, etching time on about 1 min) and to avoid any modification of this layer during the following steps. After, the actuator beam consisting of a low-pressure chemical vapour deposition Si_3_N_4_ was deposited at 800 °C on top of the SiO_2_ layer, resulting in large tensile internal stress, and patterned by classical photolithography. The thickness of the Si_3_N_4_ actuator material is related to the Pd film thickness. Here the actuator layer thickness was 30±2 nm for a film thickness of 90±5 nm. The average mismatch strain in the Si_3_N_4_ actuator material is equal to −0.0029 (see ref. 26 for details about the test structures used to estimate it). The Young’s modulus was directly measured by nanoindentation as equal to 240±10 GPa. Similar values were obtained using another method^27^. Then, the Pd film has been evaporated at 1 Å s^−1^ on a 1-nm-thick Ti adhesion layer and on top of the former layers. The Pd layer and its adhesion layer have been patterned using lift-off photolithography. A reversal photoresist was used for the lift-off. The photoresist was first spin-coated and baked at 110 °C during 90 s. Then, it was exposed using the appropriate mask and re-baked at 120 °C during 90 s. Afterwards, a last exposure without mask was performed to inverse the resist. Finally, the resist was developed in a tetramethylammonium hydroxide-based solution. To pattern the backside of the wafer, spin-coating and -patterning of a plasma-resistant photoresist was performed. This step is followed by a deep reactive ion etching from the backside of the Si wafer until reaching the SiO_2_ layer. The design of etched area is such that 3 mm in diameter rings are created with an opened area in the middle where the tensile structures are free-standing (Fig. 1a). Then, the wafers were cleaned with acetone and methanol to remove all photoresist residues.

To facilitate the later microscopic observations and to concentrate the deformation, notches have been machined every two beams with FIB (see details further). Notches have a disc shape and reduce the beam width by 40% (Supplementary Fig. 10). Still, the tensile structures are lying on the sacrificial layer. The release of the test structures was performed at room temperature by etching the SiO_2_ sacrificial layer with HF (73%). Prior etching selectivity studies performed with the same conditions have shown almost no alteration of the Si_3_N_4_ and Pd layers by the HF etchant. The tensile structures are now free-standing and directly ready to be observed in the TEM. Once the structures are released, plastic deformation takes place. It is thus no longer possible to observe the initial (that is, undeformed) microstructure of the films used in the creep experiments in TEM. TEM cross-section thin foils have thus been prepared by FIB on the as-deposited films in order to observe *ex situ* the initial microstructure (that is, grain size, dislocation density, coherency of the growth TBs and so on). Once released, the structures were subjected to a critical point drying treatment in order to avoid lateral stiction. Measurements of the displacement *u* and of all geometric parameters were carried out in a FEG SEM. Several cursors are located on the sides of the structures. Four measurements are performed for each structure (on each side of the cursor and using at least one cursor on each side of the beam). All structures are labelled based on the position on the wafer. This labelling is important for relaxation measurement to ensure following exactly the same structure each time. Ellipsometry was used to measure the actuator and sacrificial layer thicknesses. A profilometer was used to evaluate the curvature of the system after each deposition (in order to estimate the internal stress using the Stoney method) and to measure the thickness of the nontransparent Pd layer.

### Mechanical analysis of relaxation

The relaxation experiment is performed by measuring deformation at different time intervals. Both stress and strain vary with time. The strain-rate sensitivity *m* (Equation 5) and the apparent activation volume *V*_*app*_ (Equation 6) (see Supplementary Note 4 for the estimation of the true activation volume based on the apparent one) can be extracted by following the stress–strain evolution of the Pd beams as


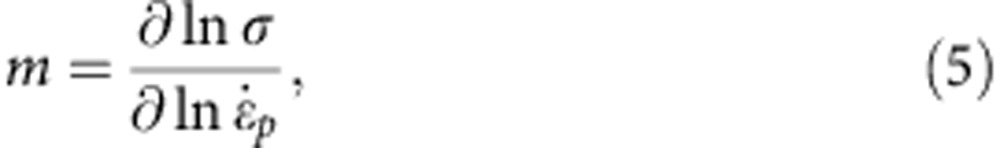



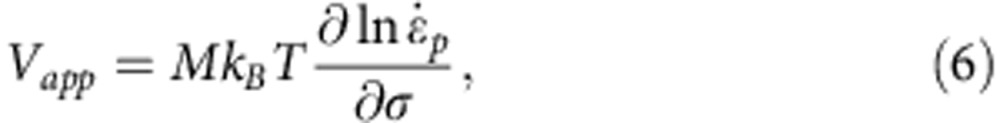


where *σ* is the stress in the beam, 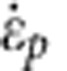 the plastic strain rate, *M* the Taylor factor, *k*_B_ the Boltzmann constant and *T* the temperature.

The experimental determination of *m* and *V*_*app*_ has been performed using two methods: a simple procedure based on some preliminary assumptions and a more complex one that has already been detailed in ref. 22 but that will be also presented hereafter.

The behaviour of one Pd beam is schematically shown in Supplementary Fig. 11. Before the release step, the stress inside the actuator is maximum. If this actuator is released alone without being attached to a Pd beam, the stress would be zero. When the system actuator sample is released, it reaches force equilibrium. As the actuator does not relax (which has been verified by measuring the displacement of test structures made of a SiN actuator and SiN specimen over long periods of time), the way this equilibrium evolves is because of the relaxation of the Pd sample (Supplementary Fig. 11).

After successive measurements of one specific structure inside an SEM, the displacement evolution with time is obtained, thanks again to the moving and reference cursors (Supplementary Fig. 8). On the basis of the variation of *u* with time, one can determine using Equations 1 and 4 the variation with time of the total strain *ε*^mech^ and stress *σ*, respectively. The plastic strain is obtained by subtracting the elastic strain (*σ*/*E* where *E* is the Young’s modulus) from the total strain. Then, the plastic strain rate is obtained by derivation with time, and finally the derivative of the plastic strain rate by the stress is performed^22^. As a first simple mean to analyse the displacements *u* versus time *t* data, a logarithmic variation is assumed. The fits are shown in Supplementary Fig. 12 for the beams analysed in the present paper. Imposing a logarithmic variation of *u* with *t* leads to a constant average activation volume independent of time.

The second fitting procedure involves more sophisticated derivation procedures based on nurbs or cubic splines to fit the *u* versus *t* data. On the basis of the fitted curve, the plastic strain and stress evolutions can be obtained and, using derivatives, one gets the strain-rate sensitivity evolution and the activation volume of this Pd beam (Supplementary Fig. 13). The procedure is repeated to compare the values of different beams with different initial stress levels (Supplementary Fig. 14). The results are scattered because of experimental errors. Nevertheless, the average level of *V* does not change. The only new information is a moderate to large increase in *V* with relaxation. Note that all these measurements are performed on straight beams. The notches produced by FIB are only used for TEM analysis. All test structures, with or without notches, come from the same wafer and are thus nominally the same.

### Preparation of notches for time-resolved TEM analysis

Notches were milled every two Pd beams using a FIB of 30 kV per 7 pA before release. The goal was to produce a shallow stress and strain concentration in a small area to facilitate local HRTEM and ACOM TEM observations. The beams without notches, that is, involving a perfectly uniaxial stress state, were used for the mechanical analysis. Supplementary Fig. 10 shows a low-magnification TEM image of a Pd beam with notches. It is worth mentioning that, most of the time-resolved HRTEM investigations (that is, dislocation density and TB-thickness measurements) were performed close to the edge of the notches. Indeed, owing to FIB milling, the thickness of these edges was smaller than in the rest of the Pd beam (~90 nm). The stress in the notched region is obtained from the original stress through correcting for the new cross-section area assuming that the machining of the notch has not changed significantly the state of equilibrium (which is correct in view of the small notch opening). The strain in the notched region can then be approximately deduced from the known stress level. Then, the corresponding strain can be obtained using the stress–strain relationship (Fig. 1b).

### Sample thinning for *ex situ* TEM and ACOM-TEM analysis

Owing to the submicron dimensions of the studied Pd beams, FIB thinning with the ‘lift-out’ procedure was used for the preparation of TEM samples from the as-deposited Pd beams for *ex situ* TEM analysis. The main advantage of this technique is that TEM samples can be made directly from the desired location. Cross-sectional and plan-view TEM thin foils were prepared using FIB. A protective Pt layer was deposited on the Pd beam before FIB milling. This prevents the surface of the Pd beam being damaged by the incident Ga^+^ ions. Ion beams of 30 kV per 3 nA and 30 kV per 0.3 nA were used for sample-cutting and early-stage milling, with a probe size of 81 and 33 nm, respectively. For the final step, an ion beam of 5 kV per 14 pA was employed to achieve the final milling and to minimize any amorphous layers generated during high-voltage FIB milling. Preparing TEM thin foils by FIB ensures the existence of one grain through the thickness that improves the HRTEM and ACOM-TEM observations.

### HRTEM analysis

HRTEM analysis (TB thickness and dislocation density) was performed in a TECNAI G2 (Field Emission Gun, 200 kV) microscope during the relaxation of the Pd films in order to follow the time evolution of the dislocation density as well as the accumulation of dislocations at the TBs. The HRTEM observations were made in the notched area of the beams to ensure the observation of a plastically deforming or relaxing zone and to provide landmarks for the follow-up of exactly the same well-oriented grains each day. The successive observations performed on different days (while keeping the sample in the holder but outside of the microscope) have been carried out carefully in nearly identical orientation and imaging conditions. Tilting the samples was thus intentionally avoided to facilitate the repeatability of the observations in similar orientation conditions. FFT patterns were recorded and their symmetry was analysed and compared. Consequently, the dislocation density as well as the TB thickness measurements do not suffer from misorientation artefacts (see Supplementary Note 3). Furthermore, the edges of the notches often contain FIB-induced amorphous domains that can be used as references for the acquisition of HRTEM images in similar defocus conditions.

The evolution of the TB thickness was performed on HRTEM images via the time-resolved observation of the coherency of growth ∑3 {111} TBs. The HRTEM images were acquired at different times from the same regions containing these boundaries. The TB thickness term is defined as the distance separating the two last nondistorted twinning planes located in the matrix grain and the twin grain. An attempt was made to determine the exact positions of these planes in [110]-oriented HRTEM images using the variation of the intensity profile along the second set of {111} planes inclined relative to the twinning plane in the IFFTs. Supplementary Fig. 15 shows an example of this approach. In this figure, the position of the last nondistorted (111) twinning plane in the matrix grain is determined from the intensity profile plotted along the (
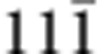
) plane in the IFFT. This position is indicated by a black arrow in Supplementary Fig. 15 in which a clear drop of the intensity is observed because of a perturbation of the stacking sequence ABCABC… inducing a shift of the position of the atoms. Such a feature is attributed to a local loss of coherency of the TB because of the accumulation of dislocations at this boundary.

The detection uncertainty of the last nondistorted twinning plane using the IFFTs corresponds to one atomic plane (see the intensity profile of Supplementary Fig. 15). By consequence, the estimated error for the measurement of the TB thickness ratio (*T*_m_/*T*_i_) is ±0.3 nm using the following formula:


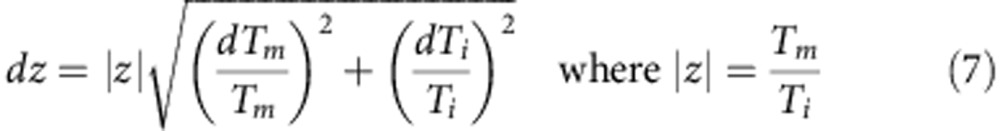


Dislocation density was measured by counting extra half planes on IFFTs generated from HRTEM images using masks applied on each *g* vector. The time evolution of dislocation density for the region indicated in Fig. 3b is presented in Supplementary Fig. 4. It can be seen that the local dislocation density measured in this region follows the average relaxation trend shown in Fig. 3a.

### ACOM-TEM analysis

The grain size distribution and the crystallographic texture of the Pd films were investigated using the ASTAR method in a Philips CM20 microscope (LaB6, 200 kV). This technique that uses small probe diffraction spot (spot of 13 nm here) patterns in TEM is an effective method for mapping phase and crystal orientation and an alternative to more conventional electron-backscattered diffraction (EBSD) in SEM based on Kikuchi line patterns. The ACOM-TEM technique has better spatial resolution (8 nm in the present case) in comparison with EBSD, which has a spatial resolution in the range of 30–50 nm depending on the material type^28^,^29^,^30^. A selected area is scanned with a small probe and the electron diffraction spot patterns are collected using an external CCD camera. Off-line, every diffraction pattern is compared with the pre-calculated templates of selected phases and the best match is selected^31^. In order to eliminate the ambiguity that can exist for highly symmetric orientations, a build in clean-up procedure in the OIM TSL software from EDAX Inc. has been used. By using this clean-up procedure, a single orientation for each ambiguous condition is selected.

ACOM-TEM orientation maps are 250 × 250 pixels in size and were obtained using a step size of 8 nm with an acquisition frequency ~70 frames per second. In order to avoid ambiguities for the measurement of the grain size distribution, Σ3 {111} TBs were excluded from the measurements shown in Fig. 2a.

To determine the grain size, histograms of grain size distribution were fitted using a lognormal distribution^32^:


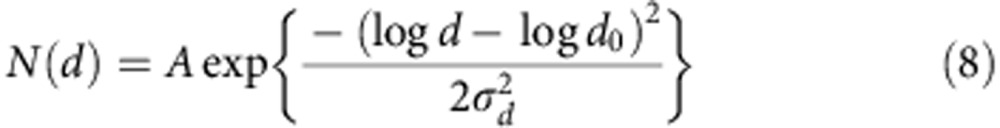


where *N(d)* is the number of grains with a particular width *d*, *A* is the amplitude of the mode, *d*_0_ is the mean of the width and *σ*_*d*_ is the s.d.

## Author contributions

M.-S.C. prepared the nanomechanical testing samples and performed the mechanical tests and analysis. B.A.-A. and H.I. performed HRTEM observations and FIB sample preparation. L.M. and S.G. performed and analysed ACOM-TEM characterizations. All authors contributed to the interpretation of the results. M.-S.C., H.I. and T.P. wrote the manuscript with contributions from the other authors. B.A.-A. and M.-S.C. prepared the figures. T.P., D.S., J.-P.R. and H.I. conceived the concept of the research. All authors commented on the final manuscript and conclusions of this work.

## Additional information

**How to cite this article**: Colla, M.-S. *et al*. Dislocation-mediated relaxation in nanograined columnar palladium films revealed by on-chip time-resolved HRTEM testing. *Nat. Commun.* 6:5922 doi: 10.1038/ncomms6922 (2015).

## Supplementary Material

Supplementary Information Supplementary Figures 1-15, Supplementary Notes 1-6 and Supplementary References

## References

[b1] Meyers M., Mishra A. & Benson D. Mechanical properties of nanocrystalline materials. Prog. Mater. Sci. 51, 427–556 (2006).

[b2] Jonnalagadda K., Karanjgaokar N., Chasiotis I., Chee J. & Peroulis D. Strain rate sensitivity of nanocrystalline Au films at room temperature. Acta Mater. 58, 4674–4684 (2010).

[b3] Pardoen T. Size and rate dependent necking in thin metallic films. J. Mech. Phys. Solids 62, 81–98 (2014).

[b4] Wang Y., Hamza A. & Ma E. Temperature-dependent strain rate sensitivity and activation volume of nanocrystalline Ni. Acta Mater. 54, 2715–2726 (2006).

[b5] Asaro R. J. & Suresh S. Mechanistic models for the activation volume and rate sensitivity in metals with nanocrystalline grains and nano-scale twins. Acta Mater. 53, 3369–3382 (2005).

[b6] Dao M., Lu L., Asaro R. J., De Hosson J. T. M. & Ma E. Toward a quantitative understanding of mechanical behavior of nanocrystalline metals. Acta Mater. 55, 4041–4065 (2007).

[b7] Nix W. Mechanical properties of thin films. Metall. Trans. A 20, 2217–2245 (1989).

[b8] Mompiou F. . Inter- and intragranular plasticity mechanisms in ultrafin-grained Al thin films: an *in situ* TEM study. Acta Mater. 61, 205–216 (2013).

[b9] Rajagopalan J., Rentenberger C., Karnthaler H. P., Dehm G. & Saif M. T. A. *In situ* TEM study of microplasticity and Bauschinger effect in nanocrystalline metals. Acta Mater. 58, 4772–4782 (2010).

[b10] Lu L. . Nano-sized twins introduce high rate sensitivity of flow stress in pure copper. Acta Mater. 53, 2169–2179 (2005).

[b11] Bergers L., Hoefnagels J., Delhey N. & Geers M. Measuring time-dependent deformations in metallic MEMS. Microelec. Reliab. 51, 1054–1059 (2011).

[b12] Kobrinsky M. J. & Thompson C. V. Activation volume for inelastic deformation in polycrystalline Ag thin films. Acta Mater. 48, 625–633 (2000).

[b13] Idrissi H. . Ultrahigh strain hardening in thin palladium films with nanoscale twins. Adv. Mater. 23, 2119–2122 (2011). 21462371 10.1002/adma.201004160

[b14] Colla M. S. . High strength-ductility of thin nanocrystalline palladium films with nanoscale twins: on-chip testing and grain aggregate model. Acta Mater. 60, 1795–1806 (2012).

[b15] Wu X., Zhu Y. T., Wei Y. G. & Wei Q. Strong strain hardening in nanocrystalline nickel. Phys. Rev. Lett. 103, 205504 (2009). 20365992 10.1103/PhysRevLett.103.205504

[b16] Wang L. . In-situ observation of dislocation behavior in nanometer grains. Phys. Rev. Lett. 105, 135501 (2010). 21230786 10.1103/PhysRevLett.105.135501

[b17] Lee J. H., Holland T. B., Mukherjee A. K., Zhang X. & Wang H. Direct observation of Lomer-Cottrell Locks during strain hardening in nanocrystalline nickel by in situ TEM. Sci. Rep. 3, 1061 (2013). 23320142 10.1038/srep01061 PMC3544074

[b18] Wang B. . Advanced TEM investigation of the plasticity mechanisms in nanocrystalline freestanding palladium films with nanoscale twins. Int. J. Plasticity 37, 140–156 (2012).

[b19] Rodney D. & Phillips R. Structure and strength of dislocation junctions: an atomic level analysis. Phys. Rev. Lett. 82, 1704–1707 (1999).

[b20] Ni S. . The effect of dislocation density on the interactions between dislocations and twin boundaries in nanocrystalline materials. Acta Mater. 60, 3181–3189 (2012).

[b21] Zhu Y., Liao X. & Wu X. Deformation twinning in nanocrystalline materials. Prog. Mater. Sci. 57, 1–62 (2012).

[b22] Coulombier M. . On-chip stress relaxation testing method for freestanding thin film materials. Rev. Sci. Instrum. 83, 105004 (2013). 23126797 10.1063/1.4758288

[b23] Caillard D. & Martin J. L. Thermally Activated Mechanisms in Crystal Plasticity Pergamon, Oxford (2003).

[b24] Gravier S. . New on-chip nanomechanical testing laboratory - Applications to aluminium and polysilicon thin films. J. Microelectromech. Syst. 18, 555–569 (2009).

[b25] Gallacher B. J., O’Neill A. G., Bull S. J., Wilson C. J. & Horsfall A. B. Analysis of a passive sensor for predicting process-induced stress in advanced integrated circuit interconnect. IEEE Trans. Device Mater. Rel. 8, 174–181 (2008).

[b26] Laconte J. . Thin films stress extraction using micromachined structures and wafer curvature measurements. Microel. Eng. 76, 219–226 (2004).

[b27] Boé A., Safi A., Coulombier M., Pardoen T. & Raskin J. P. Internal stress relaxation based method for elastic stiffness characterization of very thin films. Thin Solid Films 518, 260–264 (2009).

[b28] Humphreys F. J. Characterisation of fine-scale microstructures by electron backscatter diffraction (EBSD). Scr. Mater. 51, 771–776 (2004).

[b29] Zaefferer S. On the formation mechanisms, spatial resolution and intensity of backscatter Kikuchi patterns. Ultramicroscopy 107, 254–266 (2007). 17055170 10.1016/j.ultramic.2006.08.007

[b30] Galceran M. . Automatic crystallographic characterization in a transmission electron microscope: applications to twinning induced plasticity steels and Al thin films. Microsc. Microanal. 19, 693–697 (2013). 23642730 10.1017/S1431927613000445

[b31] Rauch E. F. & Veron M. Coupled microstructural observations and local texture measurements with an automated crystallographic orientation mapping tool attached to a TEM. J. Mater. Sci. Eng. Tech. 36, 552–556 (2005).

[b32] Söderlund J., Kiss L. B., Niklasson G. A. & Granqvist C. G. Lognormal size distributions in particle growth processes without coagulation. Phys. Rev. Lett. 80, 2386–2388 (1998).

