# Supplementary material for: Dislocation-mediated relaxation in nanograined columnar palladium films revealed by on-chip time-resolved HRTEM testing
Source: Nat Commun. 2015 Jan 5;6:5922. doi: 10.1038/ncomms6922 (PMC4354052; doi:10.1038/ncomms6922)
Supplement: Supplementary Information — Supplementary Figures 1-15, Supplementary Notes 1-6 and Supplementary References [file ncomms6922-s1.pdf]

Supplementary figures

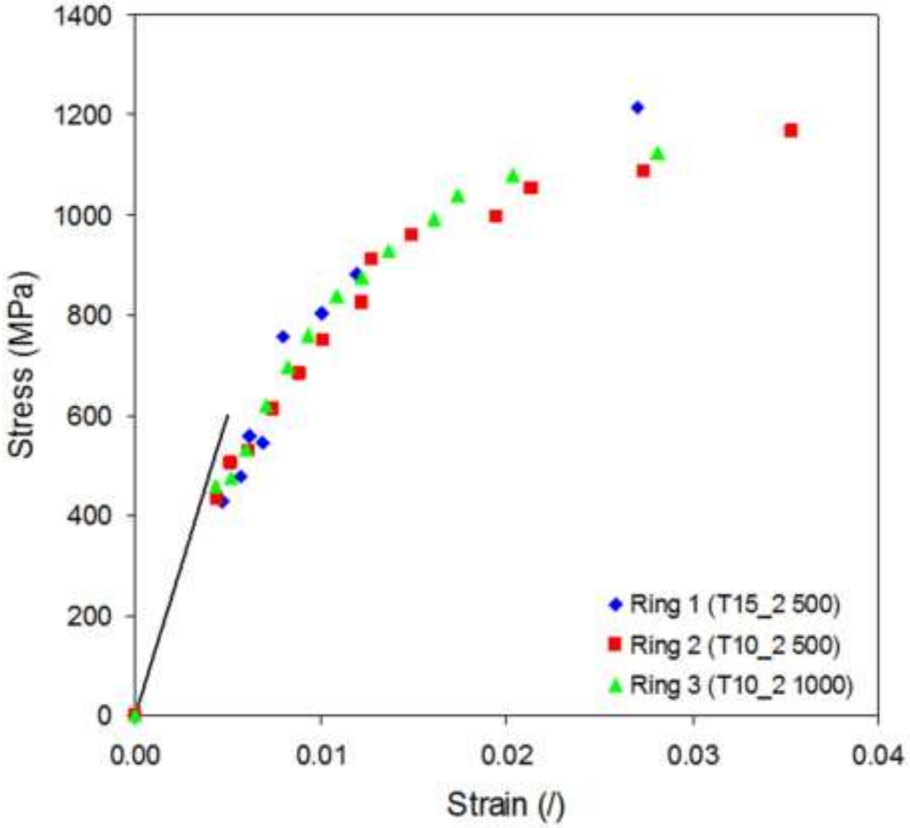

Supplementary Figure 1. Comparison of three different series of structures coming from the same batch. Each series has been measured directly after release of three different rings containing the structures designed for TEM observation, involving thus negligible relaxation. The curves are overlapping, showing the reproducibility of the technique.

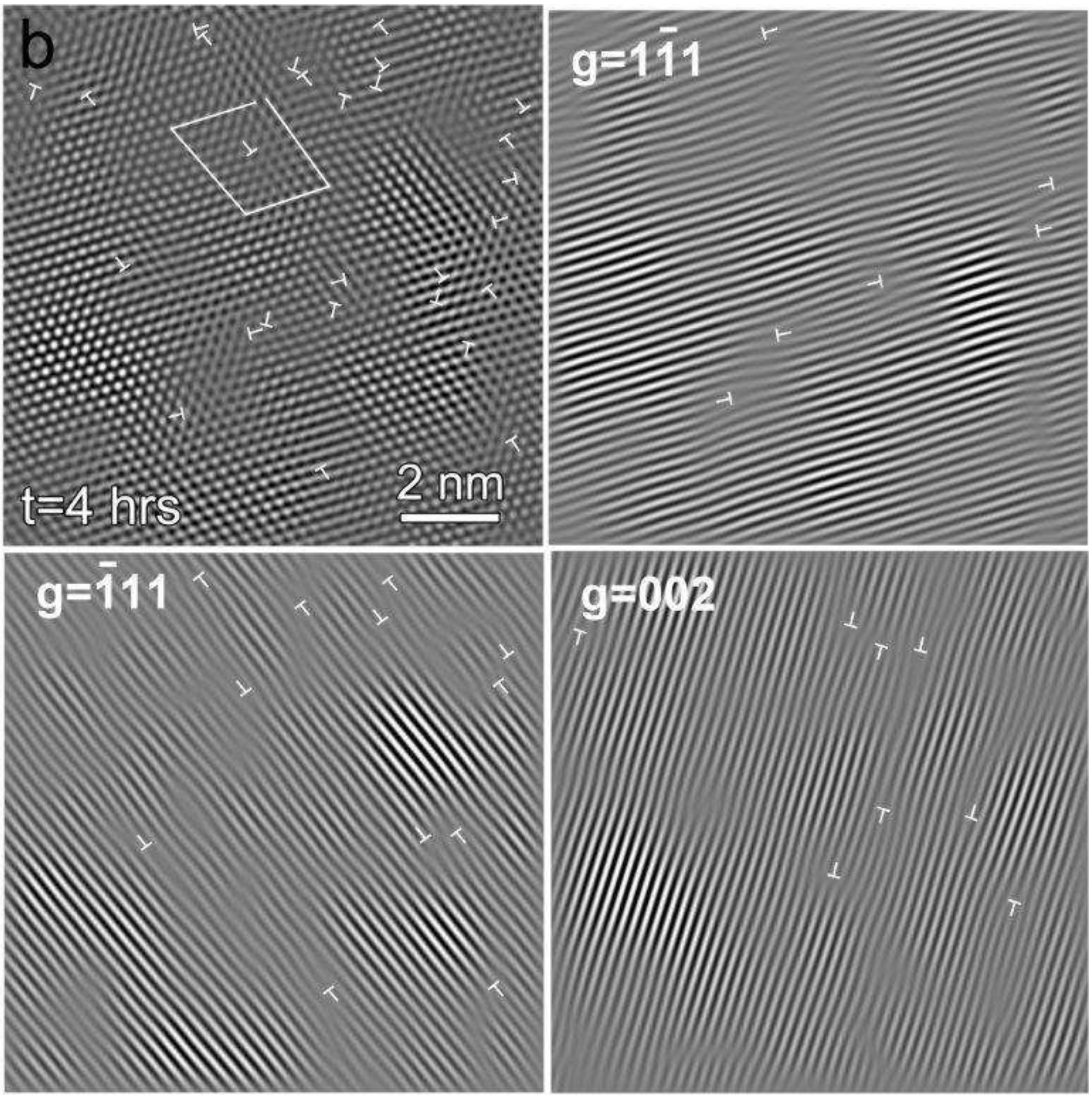

Supplementary Figure 2. Determination of dislocation sites in a single HRTEM image via ending fringes in three IFFT images (example Fig. 3b).

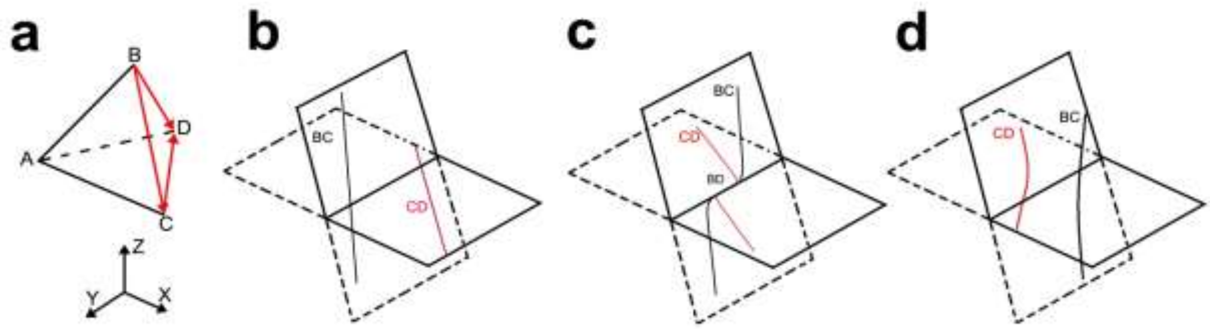

Supplementary Figure 3. (a) Thompson tetrahedron showing the Burgers vectors of the interacting dislocations. (b) and (c) formation of Lomer-Cottrell lock BD due to the interaction of two perfect dislocations BC and CD. (d) Destruction of the Lomer-Cottrell dislocation at high stress.

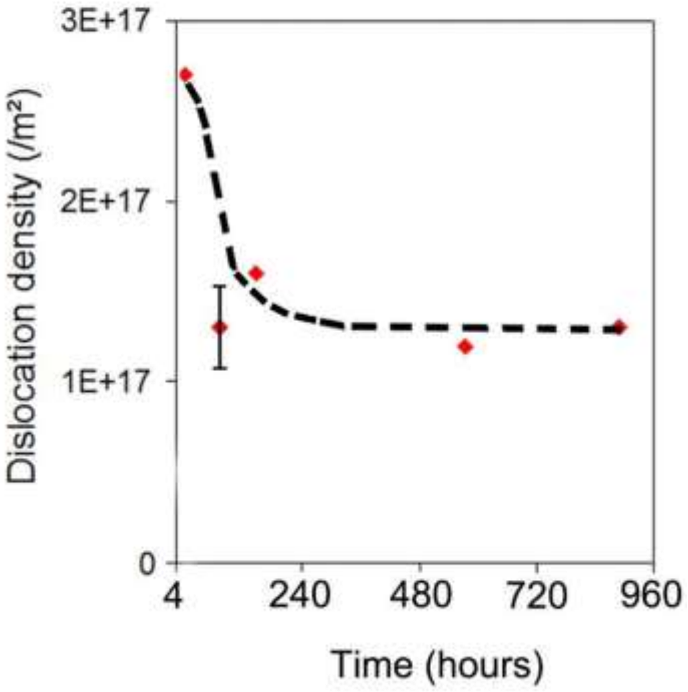

Supplementary Figure 4. Time evolution of the dislocation density measured for the region presented in Fig. 3b to d.

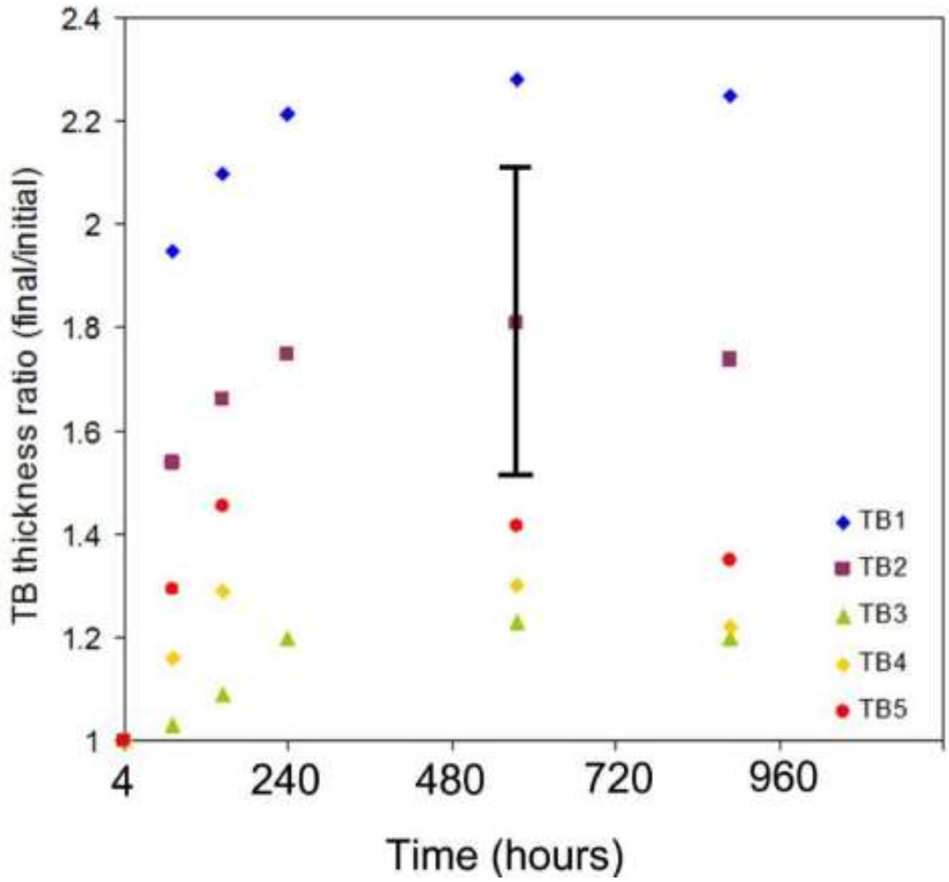

Supplementary Figure 5. Time evolution of the TBs thickness ratio after release (the first measurement was taken 4 hours after release). Similar behaviour can be seen for all the observed TBs. The error for the measurement of the TBs thickness ratio is ± 0.3.

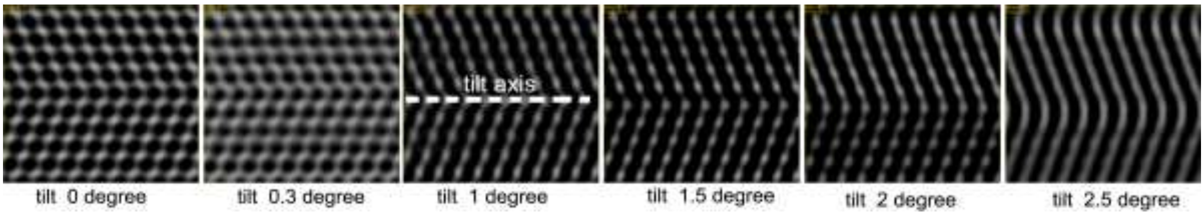

Supplementary Figure 6. Multislice simulation of the effect of a misorientation between the electron beam and the <110> zone axis on the HRTEM contrast of Σ3 {111} coherent TB. Foil thickness = 86.7 nm and defocus = 15.27 nm.

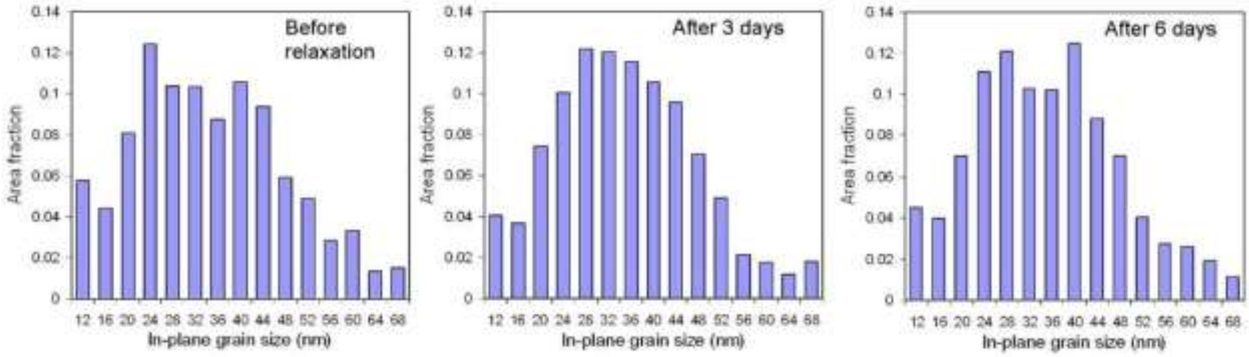

Supplementary Figure 7. Grain size distribution of a Pd beam initially deformed at 0.6%. The thickness of the beams under tensile stress coming from on-chip nanomechanical testing was not reduced using FIB. This renders the orientation indexation impossible using ACOM-TEM. However, thanks to the columnar structure, the extraction of the grain size distribution evolution upon relaxation was possible. The average grain size is equal to 23.6±11 nm before relaxation (right after the first applied deformation) and 25.2±11 nm and 24.8±11 nm after 3 and 6 days respectively. The variation of the average grain size is below the spot size used in the scan (~8 nm). The grain size can consequently be considered as constant upon relaxation which confirms the absence of grain growth during relaxation. The same observation is made on other beams with different initial strain levels before relaxation.

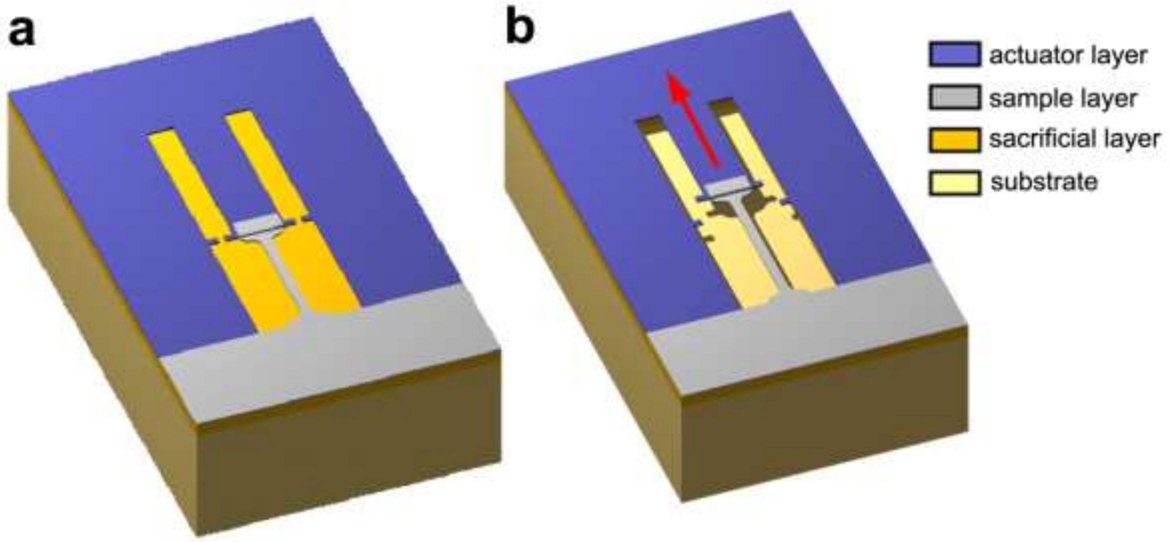

Supplementary Figure 8. Elementary internal stress actuated tensile test structure, (a) before release, (b) after release.

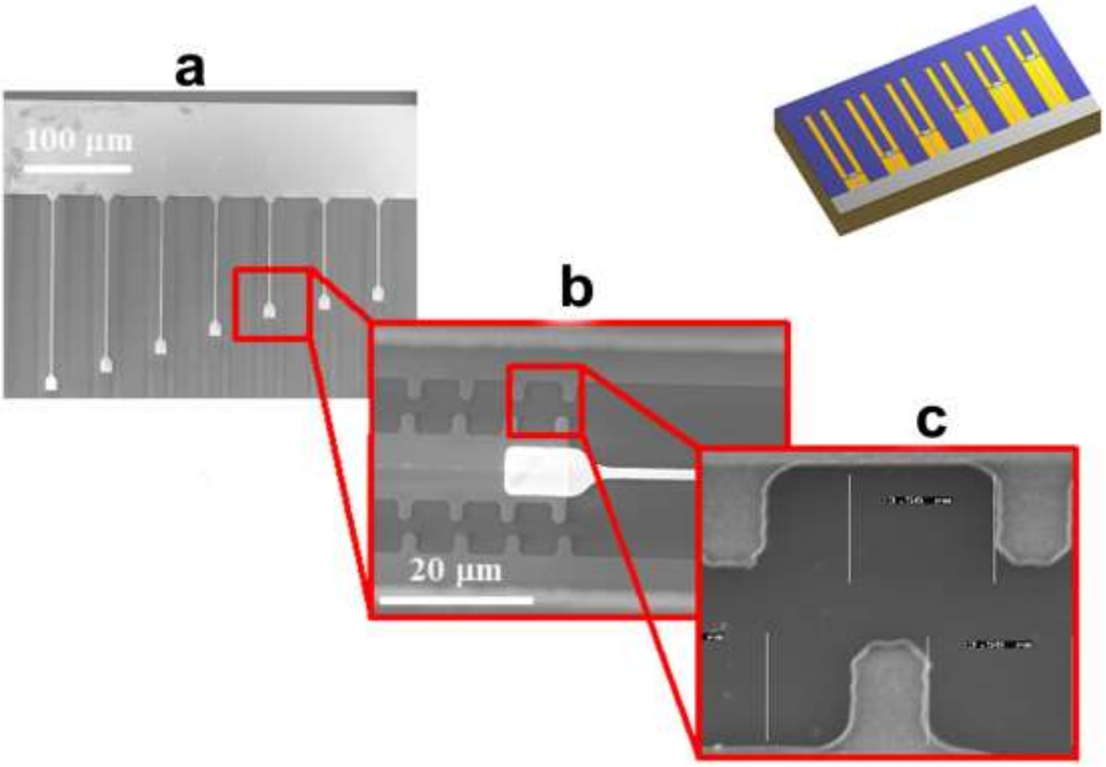

Supplementary Figure 9. Implementation of the internal stress based test structures; (a) set of tensile test structures with various actuator and specimen lengths used to generate a full stress-strain curve; (b)-(c) zoom on the cursors used to measure the displacement applied to the test specimen.

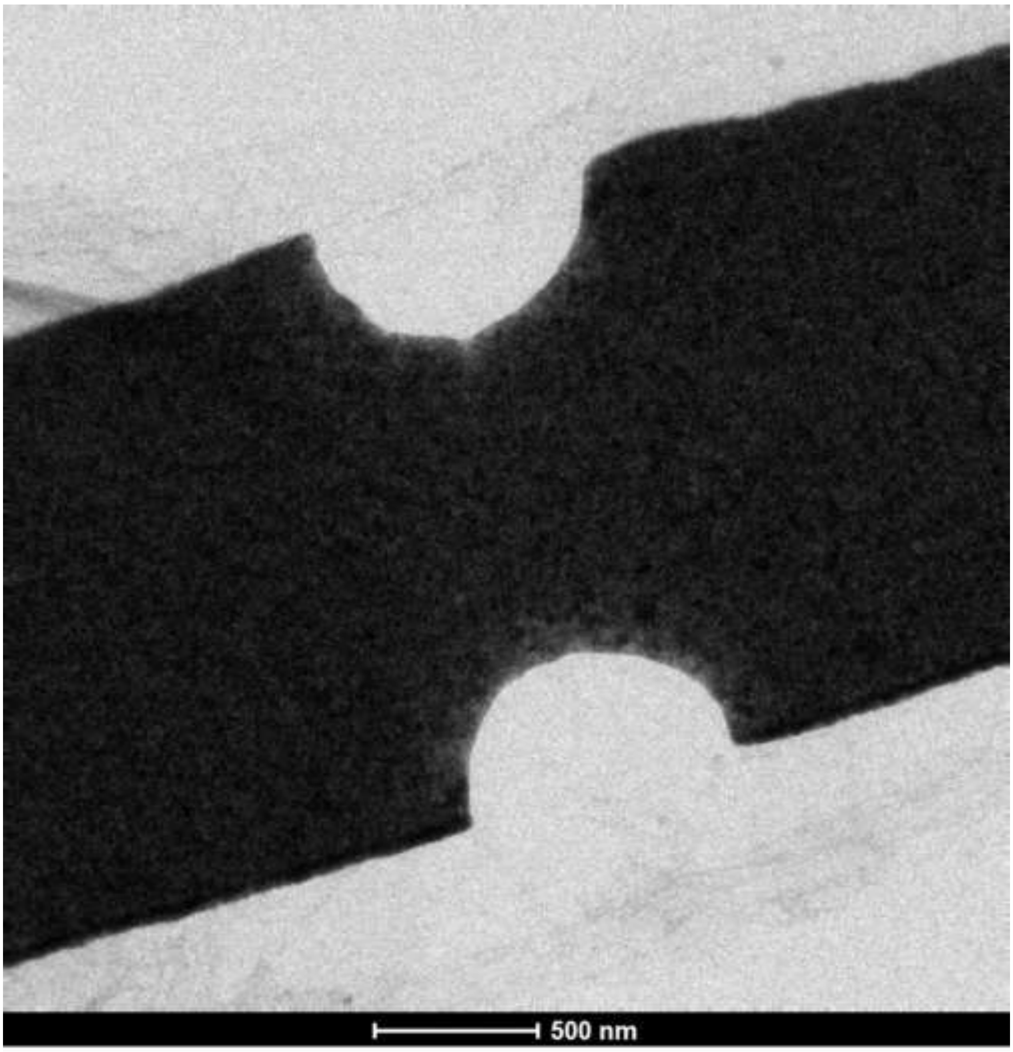

Supplementary Figure 10. Low magnification bright field TEM micrograph of a Pd beam with notches machined by FIB.

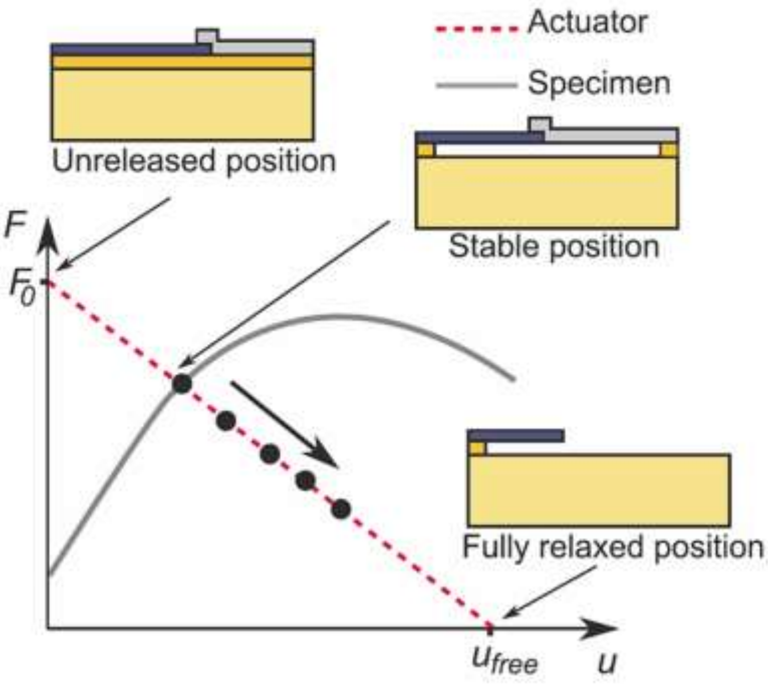

Supplementary Figure 11. Force-displacement diagram for the actuator and the specimen beams with equilibrium point attained during the release and a few relaxation points. Figure taken from Ref. 6.

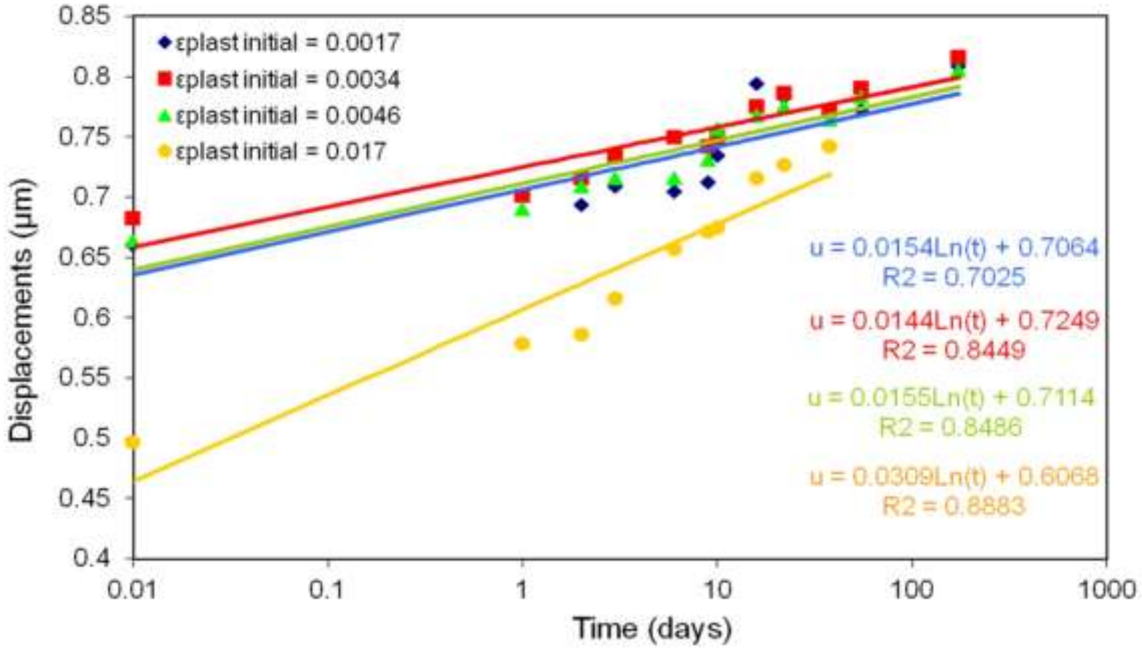

Supplementary Figure 12. Logarithmic fitting of the displacement vs. time data. The repeated wavy shape of the displacement curve for the different analysed beams comes from the systematic error made during the measurements. This error depends on the measurement day and not on the measured beam, and can be evaluated as equal to 20 nm.

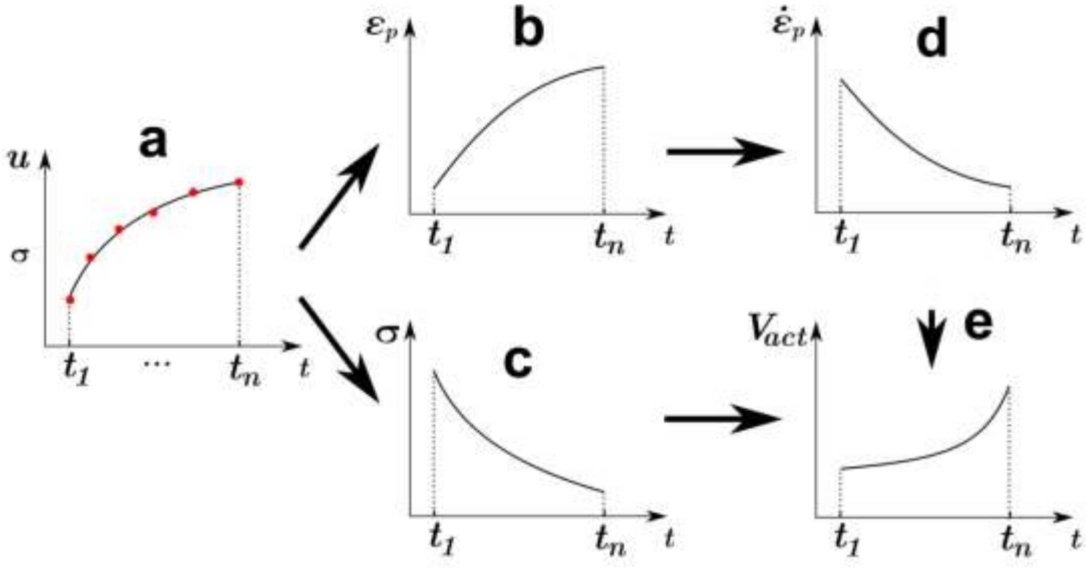

Supplementary Figure 13. Methodology for the extraction of the strain rate sensitivity parameters from a specific test structure. Figure taken from Ref. 6.

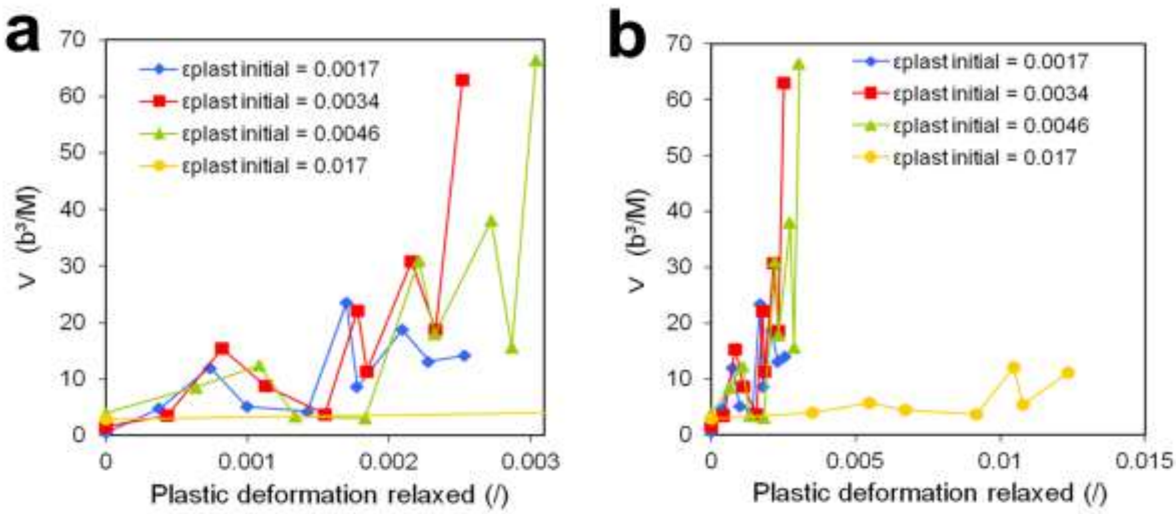

Supplementary Figure 14. (a) Evolution of the activation volume *V* with respect to the amount of relaxed deformation obtained using the second extraction procedure involving splines to fit the *u* vs. *t* data. (b) Zoom on the small displacement part of (a).

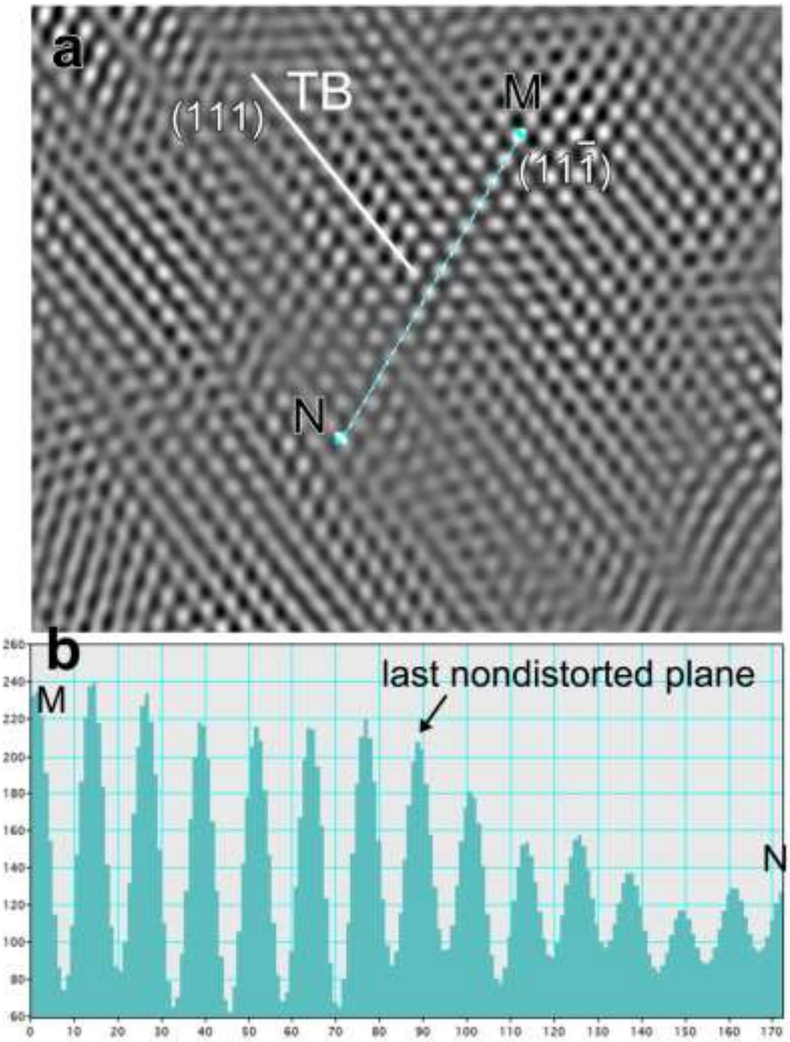

Supplementary Figure 15. (a) IFFT of HRTEM image obtained on Pd film relaxed after 36 days. (b) Intensity profile plotted from "M" to "N" along the $(11\bar{1})$ atomic plane in (a). The position of the last non-distorted twinning plane is indicated by a black arrow.

Supplementary Notes

Supplementary Note 1- Counting dislocations in HRTEM images

The method for counting the dislocations is based on a number of [110] HRTEM images of selected regions taken at different defocus. From these 2D HRTEM images IFFT images with $\vec{g} = 1\bar{1}1$, $\vec{g} = \bar{1}11$ and $\vec{g} = 002$ are produced from which sites with edge dislocations can be recognized as ending fringes by following the 1D lattice fringes at a grazing incidence. An example of this is shown in Supplementary Fig. 2 for the case of Fig. 3b where all such clear sites are indicated by the dislocation *T* symbol (except for those in a lower horizontal strip of appr. one-and-a-half cm of the image since this region becomes covered by the labelling). However, due to the effects of the changes of defocus and image delocalisation on the actual observed configuration of lattice fringes, not all dislocations are recognized as such in every HRTEM image. In other words, one needs to look at a series of HRTEM images from a given region but obtained at different defocus to get a complete picture of the dislocations present. The precision of dislocation density measurement was calculated by enlarging or shrinking the selected region in a given HRTEM image as long as the number of dislocations does not change (the estimated uncertainty from counting too few (not observed) or too many (double counting) dislocations is found to be much lower).

Supplementary Note 2 - Mechanisms for the formation and destruction of the L-C locks

Supplementary Fig. 3 shows a schematic illustration of the formation and destruction process of the Lomer-Cottrell (L-C) lock under applied stress. First, dislocations nucleate from grain boundaries and/or of intragranular sources. When two perfect dislocations with Burgers vectors $\overrightarrow{BC}$ and $\overrightarrow{CD}$ lying respectively in the ABC and ACD slip planes glide and intersect under the applied stress, a L-C sessile junction may form following the reaction: $\overrightarrow{BC} + \overrightarrow{CD} \rightarrow \overrightarrow{BD}$.

The resulting Burgers vector $\overrightarrow{BD}$ is not contained in the two initial glide planes. Therefore, the L-C dislocation is of sessile type and could act as strong barriers for glissile dislocations increasing thus the strain hardening capacity. However, these locks can be destroyed when a critical applied stress is reached as supported by recent MD simulations[1]. Supplementary Fig. 3d shows the unzipping of the L-C lock following the reaction: $\overrightarrow{BD} \rightarrow \overrightarrow{BC} + \overrightarrow{CD}$. The length of the L-C segment decreases with increasing stress resulting in the junction breaks under high stress.

Supplementary Note 3 – TB thickness evolution for different TB

The HRTEM measurement of {111} TBs thickness was performed in nano-grains exhibiting different grain sizes and local orientations. Supplementary Fig. 5 shows the TB thickness evolution versus time including the TB1 and TB2 of Fig. 4 in the manuscript. The two groups (TB1, TB2, TB3) and (TB4, TB5) were observed in two different grains. In the graph of the Supplementary Fig. 5, it can be seen that all the {111} TBs are following the same trend with a clear increase of the TBs thickness at the first stage of relaxation followed by a steady state. It is worth noting that nano-grains exhibiting different local orientations will develop different amounts of plastic deformation with different numbers of activated slip systems and nature of dislocations interacting with the TBs. This could explain the variation of the TBs thickness ratio in Supplementary Fig. 5. The TBs thickness may also vary in one individual nano-grain as can be seen for example for TB1, TB2 and TB3. This can be explained by taking into account the position of the TBs relative to the active dislocation source in one nano-grain.

It is worth justifying that the distortions observed at the TBs shown in Figure 4 cannot be attributed to a slight misalignment of these boundaries relative to the electron beam. Although the FFTs of the images in Figure 4 indicate a very small misalignment, this is insufficient to account for the observed blurring. This is indeed confirmed in the Supplementary Fig. 6, exhibiting multislice simulations of sharp and coherent Σ3 {111} TBs with an electron beam parallel to the <110> zone axis as well as with slight misorientations (up to 2.5°) around the <112> trace of the TB (note that such a choice of tilt axis can be expected to yield the most severe imaging effect). In this figure, it can be clearly seen that, as long as the atomic columns are resolved away from the boundary (up to 1.5°), a sharp twin boundary remains visible. However, for higher misorientation angles, fringe contrast

dominates the matrix image and it becomes more difficult to define precisely the position of the TB. In Figure 4, the atomic columns are clearly resolved on both sides of the TBs, indicating that the blurring of the image at the TB does not result from a slight misorientation of the boundaries relative to the electron beam. Similar conclusions were obtained for tilt axes inclined w.r.t. the TB.

Supplementary Note 4 - Apparent versus true activation volume

The apparent activation volume and the physical activation volume can significantly differ[2]. The apparent activation volume can be directly determined based on the experimental results. In order to make a semi-quantitative link with the phenomena observed by HRTEM, it is important to evaluate the correction needed to convert the apparent value into the true physical activation volume. For this purpose, we have to build a specific model which incorporates an assumed mechanism of relaxation and which must be ultimately assessed by comparing the predictions with the experimental data.

The starting point is that the in-situ TEM observations, involving dislocation density measurements, combined with the fact that no change in the grain size distribution is observed, unambiguously points to a relaxation mechanism dominated by dislocations. In the case of a dislocation controlled mechanism, the plastic strain rate is the result of the glide of the mobile dislocations with density ρ_m each one carrying a displacement b at a velocity v, i.e. (so-called Orowan equation)

$$\dot{\varepsilon}^p = \frac{\rho_m b v}{M} , \tag{1}$$

where the Taylor factor M is used to convert the average shear strains over the different slip planes into a macroscopic tensile strain. In nanocrystalline materials, the average velocity of dislocations is essentially dominated by the time to nucleate new dislocations and/or to unpin dislocations from obstacles, and not by the travelling time[3]. In the present nc Pd films, dislocations nucleate at grain boundaries and are pinned by several types of obstacles: ledges on GB, other dislocations and twin boundaries. We know from in situ observations that dislocations do indeed nucleate and do indeed travel through the grains, meaning that they are

able to escape from all these obstacles. But one (or perhaps two of these) mechanism(s) dominate over the others to set the relaxation rate. The analysis of the activation volume is a signature of the dominant mechanism. The dislocation velocity writes

$$v = \frac{\lambda}{t_{free}} \ , \tag{2}$$

where t_{free} is the time to liberate the dislocation from the "strongest obstacles" and λ is the spacing between obstacles (i.e. the grain size in the absence of other dislocations or the mean free path between pinning points). The mechanism of liberation of the dislocations from small range interactions is a thermally activated mechanism which can be described using a reaction rate type theory

$$t_{free} = t_0 \exp\left(\frac{\Delta F_0 - \sigma^{th} V}{M k_B T}\right) \ , \tag{3}$$

where t_0 involves the vibration frequency, ΔF_0 is the Helmholtz free energy to overcome the obstacle, V is the physical activation volume, and σ_{th} is the effective stress. The effective stress is the "thermal" component of the total stress which works against the short range interactions and is obtained as

$$\sigma^{th} = \sigma - \sigma_{long\_range\_stress} \ , \tag{4}$$

where $\sigma_{long\_range\_stress}$ is the a thermal contribution corresponding to the long range internal stress working against plastic flow (also called back stress).

By combining equations (2), (3) and (4), one gets

$$V = Mk_B T \frac{\partial \ln \dot{\varepsilon}^p}{\partial \sigma^{th}}\bigg|_T + Mk_B T \frac{1}{\rho_m}\frac{\partial \rho_m}{\partial \sigma^{th}} = \underbrace{Mk_B T \frac{\partial \ln \dot{\varepsilon}^p}{\partial \sigma}\bigg|_T}_{V_{app}} \frac{\partial \sigma}{\partial \sigma^{th}} + Mk_B T \frac{1}{\rho_m}\frac{\partial \rho_m}{\partial \sigma}\bigg|_T \frac{\partial \sigma}{\partial \sigma^{th}} \qquad (5)$$

The apparent activation volume V_{app}, as defined in eqn (5) is equal to V only if (i) the applied stress σ is equal to the thermally activated component σ^{th} and if (ii) the mobile dislocation density is kept constant, which means that the microstructure does not change. The stress dip test method[3] is a method from which σ^{th} can be extracted while avoiding microstructure changes. Here, the test method is such that the microstructure changes during relaxation and such that it does not allow separating σ^{th} from σ.

Hence, some further assumptions are needed to address these two difficulties. First, it is known that in nanocrystalline materials, long range back stresses are indeed significant. They can account for more than half of the flow stress, see e.g. for nc Cu[2] where it is on the order of 1/2, and sometimes even more. In nc Ni[4] with similar grain sizes as in our Pd films, σ^{th} accounts for 30% of σ only while preserving a constant ratio during deformation. In other words, $\frac{\partial \sigma}{\partial \sigma^{th}}$ can be on the order of 2 to 3.

The change of mobile dislocation density appearing in the second term of the right hand side of equation (5) is difficult to estimate precisely. If we assume that the deformation during the relaxation step is essentially carried by the existing dislocations stored in the grains at the onset of the relaxation, then the mobile dislocation density should be directly proportional to

the total dislocation density : $\rho_m = k\rho$ where k is an unknown constant. The data of the Supplementary Fig. 4 and of the Fig. 3a show that the dislocation density decreases by a factor 2 within a few days while the plastic strain rate changes by the same factor within about 1 day. Hence, one gets that

$$\left.\frac{1}{\rho_m}\frac{\partial \rho_m}{\partial \sigma}\right|_T << \left.\frac{\partial \ln \dot{\varepsilon}^p}{\partial \sigma}\right|_T = \left.\frac{1}{\dot{\varepsilon}^p}\frac{\partial \dot{\varepsilon}^p}{\partial \sigma}\right|_T .$$

In other words, with the set of assumptions made above, one can justify that the physical activation volume is about 2 to 3 times the measured apparent activation volume, perhaps slightly less if the change of mobile dislocation density is larger than expected. For the rest of the analysis, we will assume that the correction to be applied to the apparent volume is equal to 2.

The mean experimental apparent activation volume normalized by M (i.e. V_{app}/M) varies from 8 b^3 at small plastic strains down to 4 b^3 at the largest plastic strain (see Fig. 1b). Taking M equal to $\sqrt{3}$, gives V_{app} varying from 14 down to 7 b^3. The true physical activation volume thus approximately ranges typically between 28 to 14 b^3.

Supplementary Note 5 - Analytical estimate of the activation volume

The activation volume for recovery, i.e. for a relaxation mechanism based on the depinning of dislocations, can be expressed by[5]

$$V = b\,\lambda\,\Delta y^{*}\ , \qquad (6)$$

where V is the (true) activation volume, b the Burgers vector, λ the distance between the pinning points and Δy^{*} the activation distance, i.e. the distance over which a dislocation has to jump to overcome the energy barrier which retains it at its pinned position. The distance between pinning points is related to the dislocation density ρ:

$$\lambda = \alpha(\rho)^{-1/2}\ , \qquad (7)$$

where α is a geometrical factor approximately varying between 1 and 2.

The HRTEM analysis has provided the variation of the dislocation density with relaxation time (Fig. 3a). By approximating the terms present in equations (6) and (7), we can obtain the range of the theoretical activation volumes related to the relaxation via dislocation glide assuming $\Delta y^{*} \approx b$ and α =2: λ = $(7\text{x}10^{16}\ \text{m}^{-2})^{-1/2} \approx 4\ 10^{-9}$ m ≈ 16b using b = 0.275 nm for Pd and thus $V \approx 20\ b^{3}$. This magnitude of activation volume is in the range of the experimental values of the average V estimated above.

Supplementary Note 6 - Approximate model for the evolution of the activation volume with relaxation

Supplementary Fig. 14 shows an increase of *V* with relaxation. The rate of increase of *V* is large for small plastic prestrain and moderate for large plastic prestrain. This evolution can be semi-quantitatively related to the evolution of the dislocation density observed experimentally. The change of *V* with plastic strain writes

$$\frac{\partial V}{\partial \varepsilon_p} = b\Delta y^* \frac{\partial \lambda}{\partial \varepsilon_p} = -b\Delta y^* \alpha \rho^{-\frac{3}{2}} \frac{\partial \rho}{\partial \varepsilon_p} . \tag{8}$$

The connection between the dislocation density and plastic strain can be derived in the following way:

$$\frac{\partial \varepsilon_p}{\partial \rho} = \frac{\partial \varepsilon_p}{\partial n} \frac{\partial n}{\partial \rho} , \tag{9}$$

where *n* is the average number of dislocations per grain. Each dislocation liberated during recovery leads to a shear strain increment equal to b/h where *h* is the film thickness and to an increment of the tensile strain equal to $b/\sqrt{3}h$ assuming a Taylor factor equal to $\sqrt{3}$. The dislocation density is related in the following way to the number of dislocations per grain:

$$\rho = n \frac{\gamma d}{\pi h d^2 / 4} , \tag{10}$$

where d is the in plane grain size and γd is the mean length of each dislocation segment. Based on equations (9) and (10), one thus gets

$$\frac{\partial \varepsilon_p}{\partial \rho} = -\frac{\pi}{4\sqrt{3}}\frac{bd}{\gamma} \ , \tag{11}$$

hence,

$$\frac{\partial V}{\partial \varepsilon_p} = \frac{4\sqrt{3}}{\pi}\alpha\gamma\frac{b}{d}\rho^{-\frac{3}{2}} \ . \tag{12}$$

Using $\alpha = 2$, $\gamma = 2$, $d = 30$ nm, $\rho = 7\text{x}10^{16}$ m^{-2} (which corresponds to a strain of about 2%) equation (12) predicts an increase ΔV by ~ 2 b^3 for a plastic strain relaxation $\Delta\varepsilon_p$ of 0.01. This change of activation volume can be compared with the corresponding variation measured experimentally for a similar large strain equal to ~ 5 b^3 (again for $\Delta\varepsilon_p$ = 0.01), see Supplementary Fig. 14a. The present prediction assumes that all liberated dislocations will effectively contribute to useful plastic deformation, but, in reality, only a fraction of them will contribute which can explain the lower predicted variation; nevertheless the comparison is satisfactory in view of the number of underlying assumptions.

Note finally that equation (12) indicates that $\partial V / \partial \varepsilon_p$ is proportional to V^3 (combining equations to relate dislocation density to activation volume). Supplementary Fig. 14a shows that $\partial V / \partial \varepsilon_p$ is about 10 times larger for small plastic prestrains compared with the largest plastic prestrain. Figure 1b shows that the average V changes by a factor 2 from small to large

strains, and thus V^3 changes by a factor 8 in agreement with the change of $\partial V / \partial \varepsilon_p$. All these first order estimates point towards a confirmation of the underlying assumptions of our analysis.

Supplementary References

1. Rodney, D. & Phillips, R. Structure and strength of dislocation junctions: an atomic level analysis. *Phys. Rev. Lett.* **82,** 1704-1707 (1999).

2. Caillard, D. & Martin, J. L. *Thermally Activated Mechanisms in Crystal Plasticity*, (Pergamon, Oxford, 2003).

3. Duhamel, C., Brechet, Y. & Champion, Y. Activation volume and deviation from Cottrell-Stokes law at small grain size. *Int. J. Plast.* **26,** 747-757 (2010).

4. Van Petegem, S., Brandstetter, S., Van Swygenhoven, H. & Martin, J. L. Internal and effective stresses in nanocrystalline electrodeposited Ni. *Appl. Phys. Lett.* **89,** 073102 (2006).

5. Argon, A. S. *Strengthening mechanisms in crystal plasticity* (Oxford Univ. Press, Oxford, 2008).

6. Coulombier, M. *et al.* On-chip stress relaxation testing method for freestanding thin film materials. *Rev. Sci. Instrum.* **83,** 105004 (2013).
